# Sophisticated use of upper limb haptic interactions during adaptive locomotion

**DOI:** 10.3389/fpsyg.2025.1648450

**Published:** 2025-10-27

**Authors:** Michael J. MacLellan, Yury Ivanenko, Priscilla Avaltroni, Francesco Lacquaniti, Francesca Sylos-Labini

**Affiliations:** 1 Department of Applied Human Sciences, University of Prince Edward Island, Charlottetown, PE, Canada; 2 Laboratory of Neuromotor Physiology, Istituto di Ricovero e Cura a Carattere Scientifico Fondazione Santa Lucia, Rome, Italy; 3 Department of Systems Medicine and Center of Space BioMedicine, University of Rome Tor Vergata, Rome, Italy

**Keywords:** sensorimotor control, haptic communication, interaction forces, quadrupedal coordination, interactive locomotion

## Abstract

Humans commonly engage in upper limb haptic interactions during bipedal locomotion, and the expansive use of our arms makes us unique compared to the quadrupeds we evolved from. Examples of these haptic interactions include walking while carrying an object, using environmental surfaces such as a railing to provide stability assistance, and holding hands while walking with another individual. These interactions may increase the complexity of our locomotor behaviors, such as when feedforward control is employed to dampen arm motion and dissipate reaction forces at heel contact to stabilize an object we are carrying. However, these interactions also increase the available sensory information in the upper limb and can be utilized to aid in locomotor adaptation. For instance, the interaction forces experienced when holding hands or during collaborative object transport can lead to an unconscious synchronization of gait patterns between the two individuals. Recent work has further suggested that upper limb haptic interactions may have clinical relevance for improving locomotion in pathological populations. This review brings a novel, integrative perspective by examining upper limb haptic interactions in locomotion across everyday, collaborative, and clinical scenarios. In particular, the review highlights the importance of studying upper limb haptic interactions from different viewpoints to gain insight into the neuromechanical control of adaptive locomotion, as well as to investigate how these interactions can be exploited for clinical use.

## Introduction

Human bipedal evolution from quadrupedal primates has facilitated the development of an extensive repertoire of upper limb movement tasks for our daily use (Marzke, 2009). Through evolution, the motor pathways associated with our upper limbs have strengthened (Lemon, 2008), and a simultaneous increase of the amount of space in the somatosensory cortex devoted to upper limb haptic sensation occurred (Kaas, 2008), which allow us to perform fine motor skills using our upper limbs with ease. It is important to note that standard human locomotion - without the addition of specific tasks for the upper limbs - involves a stereotypical antiphase arm swing trajectory, that is likely controlled by neural networks largely inherited from evolution including quadrupeds (Dietz, 2002; Grillner, 2011; Zehr et al., 2016). When performing upper limb tasks during locomotion, haptic information is available which can subsequently be useful in superimposing the execution of these skills within our basic locomotor pattern (Ivanenko et al., 2005). This includes signals from mechanoreceptors that provide information related to the interaction between our hand and the surface we are touching, muscle spindles indicating the positioning and movement of our upper limb, and Golgi tendon organs informing us of the muscle forces used during these interactions. This information can subsequently be used to inform feedforward control of upcoming movements, or for execution of movements in response to feedback, both of which are control mechanisms that are fundamental in adapting our locomotor patterns in response to upper limb haptic interactions.

This review will focus on upper limb tasks that are performed during locomotion and the haptic interactions associated with these behaviors. The tasks we will focus on include carrying of an object during locomotion (object transport), pairs of individuals carrying an object (collective object transport), hand holding (human-human interactive locomotion), and robots designed for guiding human locomotion (human-robot interactive locomotion), all of which are forms of adaptive locomotion (i.e., the modification of walking patterns in response to individual, environmental, or task constraints). This research will highlight how information from haptic interactions can be used to facilitate feedforward locomotor control, as well as provide non-verbal cues leading to adaptation of locomotor patterns. These haptic interactions have clinical implications and provide complementary methods for clinicians to reveal impairments (i.e., for diagnostic purposes) as well as to rehabilitate spatiotemporal gait characteristics and adaptive locomotion.

## Use of haptics for control of object transport

Object transport in humans can take many forms, from the relatively simple task of carrying a backpack (Huang and Kuo, 2014) to more complex head-supported load carrying tasks as seen in Nepalese transporters (Bastien et al., 2005). A unique characteristic of human locomotion is our ability to move around bipedally, allowing us to manipulate and control objects with our upper limbs as we walk. Humans frequently walk while simultaneously carrying an object (such as a cup of coffee), and more recently, walk while manipulating a cellular phone. Right after toddlers begin walking independently, they prefer to do so while carrying objects than with hands free, despite this added task would seemingly increase the complexity of motor coordination (Heiman et al., 2019). Chimpanzees have been observed carrying objects during locomotion, however, these actions are predominantly performed in a quadrupedal-tripedal manner (Carvalho et al., 2012).

**FIGURE 1:**
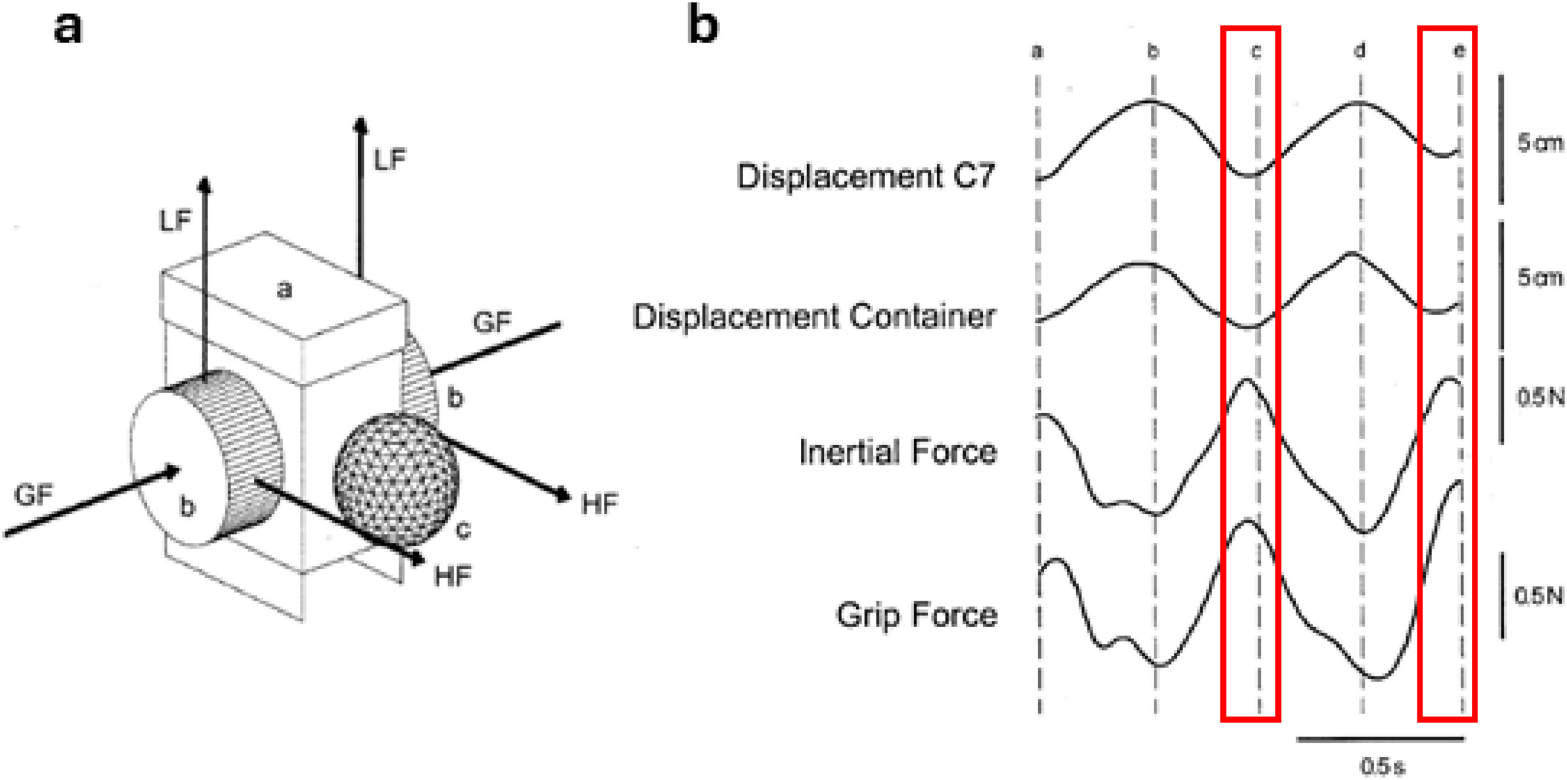
Vertical trunk-object trajectories and forces during object transport. **(a)** Transported object consisting of a plastic container with a lid (a), force transducers at each side (b), and a reflective marker (c) attached to the front. The arrows indicate the directions of the grip forces (GF), load forces (LF), and horizontal forces (HF). Inertial forces were calculated from the load and horizontal forces at each side. **(b)** Data from a representative participant illustrating the vertical trajectories of the trunk (C7 vertebrae), a container held in-hand, as well as the vertical inertial force and grip force on the container during a stride cycle. Heel contacts occur at points a, c, and e, while midstance occurs at b and d. Highlighted in red is the window around heel contact, where it can be seen that peak grip forces occur in a feedforward manner prior to contact. Figure adapted from Gysin et al. (2003) with permission.

A fundamental requirement for carrying an object during locomotion is the generation of appropriate arm and finger forces to maintain the position and orientation of the object we are holding: think of walking with a cup of coffee (Mayer and Krechetnikov, 2012). Each contact our foot makes with the ground generates a significant force that is transferred upwards through our body segments and ultimately acts at the junction between our hand and the object (Gysin et al., 2003, 2008). This upward force occurs simultaneously with a downward inertial force of the object occurring from the sinusoidal vertical motion of our body with each stride we take (Gysin et al., 2003, 2008). Object transport then requires the appropriate generation of normal forces that are applied by the fingers to stabilize the object and prevent it from slipping or changing orientation. Using an instrumented object for transport, Gysin et al. (2003) determined that the peak grip force lagged the downward peak inertial force of the object by less than 30 ms, which is shorter than the time required for a feedback-controlled motor response (Figure 1). These observations led to the conclusion that feedforward control mechanisms are utilized to generate the appropriate grip force to counteract the inertial forces of the object (Gysin et al., 2003). This is a form of anticipatory control, whereby the central nervous system regulates movement by anticipating future events as opposed to reacting after they occur. Muratori et al. (2006) determined that anticipatory grip forces are appropriately scaled when transporting objects of different mass 0.25 m from a seated position. It is possible that a similar feedforward control mechanism is used when transporting objects of different mass when walking in order to prevent slippage of the object in response to inertial forces. Moreover, the trunk and object vertically oscillate in a coupled sinusoidal pattern when carrying an object, with the lowest point of the trunk trajectory coinciding with heel contact. When carrying loads of greater mass, the vertical oscillations of the trunk tend to be maintained (Crowe et al., 1993), however, this coincides with a reduction in trunk flexion/extension range of motion (Gsell et al., 2018), which may be facilitated with feedforward control. A damping ratio that relates the vertical range of motion of the trunk and object oscillations has been used to quantify the relationship between these trajectories. This ratio is affected by the modification of mechanical stiffness in the arm, acting to increase flexion at the minimum of the vertical trunk trajectory and decreasing flexion at the highest point (Albert et al., 2010; Song et al., 2020). For instance, when carrying an unstable object such as a container of water, the vertical movement of the object decreased relative to the movement of the trunk, suggesting an adjustment of arm stiffness to maintain the position of the container to avoid spillage (Gysin et al., 2003). The muscle activities responsible for these arm actions appear in a proximal to distal pattern and initiate prior to heel contact (Song et al., 2020), strengthening the argument supporting feedforward mechanisms controlling arm motion during object transport.

Object transportation can also be viewed in the context of internal models or internal representations of whole-body motion and environment. Locomotor body schema in primates may incorporate handheld objects, tools, footwear, and support surface positioning (Iriki et al., 1996; Ivanenko et al., 2011; Pearson and Gramlich, 2010), and the presence of a hand-held object may also be integrated within this schema. Since internal models are likely used in the execution of feedforward control, the haptic information gained from the interaction with the held object is likely of the utmost importance to inform this model (Gysin et al., 2003), and it can be used in one’s locomotor body scheme (Ivanenko et al., 2005) to superimpose upper limb force production onto our locomotor pattern. Albert et al. (2010) postulated that the coupling of grip and inertial force generated by self-motion in a predictive manner very likely involve processing from the cerebellum. They also suggested the involvement of the anterior cingulated cortex, lingual gyrus, and caudate nucleus (Albert et al., 2010; Boecker et al., 2005). This anticipatory control is rather robust and observed when walking at differing velocities (Gysin et al., 2003), locomotor tasks (Diermayr et al., 2008; Gysin et al., 2008), in older adults (Diermayr et al., 2011; Gysin et al., 2008), and select neuromotor pathologies (Albert et al., 2010; Prabhu et al., 2011). Moreover, object transportation has been shown to consume attentional resources which likely are needed to appropriately integrate this sensory information within our walking pattern (Gysin et al., 2008; Oh-Park et al., 2013).

Finally, object transport requires coordination between the upper and lower limbs in order to provide appropriate upper limb stability to prepare for lower limb contact. For instance, grasping an object during walking requires a superposition of the voluntary movement upon the locomotor pattern, and a corresponding alignment of the two motor programs (Ivanenko et al., 2005). A tight coupling of the vertical trajectories of the trunk and object are present during object transport, which are controlled using feedforward control mechanisms, likely formed using the haptic information gained from the interaction with the held object. These actions work to dampen the object in preparation for inertial forces occurring at heel contact, and transport requiring an accuracy constraint require the arm to reduce motion of the object in relation to the trunk.

## Transmission of haptic information through collective object transport

Not only do humans carry objects, but an important functional example of transport is when it is performed in a collective pair, adding a social component to the mechanical task. This results in an interactive locomotor pattern that must incorporate the collective relationship between neural circuity and biomechanical action within the pair. Collective object transport is generally performed in one of two methods: either in a “side-by-side” configuration or a “one person behind the other.”

Mechanically, side-by-side collective transport of a relatively light object (∼10% of collective participant mass) has little effect on the oscillation of the vertical center of mass trajectory when compared to walking alone (Fumery et al., 2019), however, collectively carrying objects > 20% participant mass results in decreases of vertical center of mass displacement and step length (Fumery et al., 2021). Interestingly, total mechanical work and rate of energy recovery does not differ between the two conditions (Fumery et al., 2019, 2021), and the vertical trajectory of the center of mass presents a more pendular-like behavior (Fumery et al., 2019) during collective transport when compared to independent walking. Moreover, rate of energy recovery increases ∼15% in a short amount of time (within three trials) of object transport (Fumery et al., 2018). Similar to individual object transport, the addition of an accuracy constraint to collective transport resulted in a 0.17 m/s decrease of collective walking velocity in the pair, as well as a decrease of the pendular-like behavior of the center of mass (Sghaier et al., 2022).

In collective object transport, haptic information is transmitted between pairs of individuals through interaction forces in the object. For instance, Lanini et al. (2018) determined that haptic interactions during a collective pole carrying task were important in distinguishing a command for acceleration or deceleration from a leader to a follower, when the latter had visual and auditory information temporarily removed. Fumery et al. (2021) concluded that haptic interactions may be used to communicate the changes in vertical center of mass and gait cycle timing between participants, in order to perform collective transport in an energetically efficient manner, as well as distinguish a leader and follower within the transporting pair. Haptic interactions are also vital form of communication during a collective carrying task in which one participant is unaware of the goal location for the carried object (Maroger et al., 2022).

In terms of lower limb locomotor adaptations, Lanini et al. (2017) observed that, when performing a one person behind the other collective transport task, over 70% of paired participants coordinated their step patterns in a manner reminiscent of locomotion coordination in quadrupeds. In this study, the participants decreased their gait cycle time, center of mass velocity, and step length when compared to independent walking, but they matched these characteristics to the individual they were collaborating with. This resulted in the spontaneous appearance of pace (41%), trot (36%), and diagonal (23%) quadrupedal step patterns in the pairs of participants. Lanini et al. (2017) concluded that haptic interactions between participants may be partially responsible for the spontaneous appearance of these coordination patterns.

It should be noted that the rigidity or compliance (i.e., the deformation or change of shape) of the transported object is likely instrumental in the quality of the haptic interaction, as a greater amount of object deformation would alter the forces transmitted between participants. This issue has been highlighted in human-robot collective transport tasks, and methods have been developed to enhance the support from the robot while also avoiding excessive deformation of the transported object (Bonci et al., 2024). The issue of object compliance could have direct influences on the dynamic patterns, as well as the spontaneous appearance of lower limb coordination patterns presented by the dyads. Future research on the effects of transported object compliance would be beneficial in determining the effects of the diminished haptic communication when transporting a deformable object.

## Human-human interaction as a form of haptic communication

Another physiologically relevant interactive locomotor task that humans commonly perform is walking hand in hand. This human-human interaction usually starts in infancy prior to the acquisition of independent locomotion (Adolph et al., 2011), and is fundamental in the development of coordinated movements we perform throughout our lives (Arabin et al., 1996). While walking with hand contact is a common situation that we naturally experience, little is known about the forces arising from these physical interactions.

One common result of haptic communication during hand in hand locomotion is that sensory information transferred between pairs may influence the walking patterns. Specifically, this task has been shown to result in an unconscious synchronization of lower limb movements between pairs (Zivotofsky and Hausdorff, 2007) and may represent a potential optimization that is communicated through upper limb interactive forces (Sylos-Labini et al., 2018). Indeed, there are occasional episodes when non-touching pairs may spontaneously synchronize their walking behavior (Felsberg and Rhea, 2021; Hajnal and Durgin, 2023; Nessler and Gilliland, 2009, 2010; Nessler et al., 2015), however, this unconscious action is much more likely to occur during hand-in-hand interactive locomotion (Roerdink et al., 2017). The occurrence of walking synchronization was originally documented by Zivotofsky and Hausdorff (2007) who performed a video analysis of people walking while holding hands and noted ∼50% of the participant pairs spontaneously coordinated their walking patterns. Using more sophisticated analysis techniques, this probability was found to be sightly lower than originally observed and that the phase relationships between pairs waxed and waned throughout walking trials (van Ulzen et al., 2008). Subsequent research has indicated the likelihood of spontaneous synchronization during walking while holding hands closer to 40% (Sylos-Labini et al., 2018; Zivotofsky et al., 2012). When hand holding leads to synchronization, pairs are more likely to present an in-phase pattern of the lower limbs as opposed to an anti-phase pattern (Sylos-Labini et al., 2018; van Ulzen et al., 2008; Zivotofsky et al., 2012). This spontaneous synchronization is thought to occur due to a “communication link” provided by the haptic interaction of the participant’s hands that transfers non-verbal cues as to each person’s movements (Zivotofsky and Hausdorff, 2007).

Interestingly, evidence suggests that the level of walking synchronization between individuals holding hands uses attentional resources. Zivotofsky et al. (2018) studied the degree of walking synchronization during the performance of a secondary task. This secondary task involved listening to a story during walking and answering questions following the walking trial, however, complexity of the secondary task was modified by having participants identify two (simple task) or four (complex task) phonemes used in the story. Zivotofsky et al. (2018) observed a 32% increase in gait symmetry from baseline walking hand-in-hand when performing the simple secondary task, but a decrease of 10% when performing the complex task. They concluded that the simple secondary task led to increased automaticity of walking, and the more complex task was attentionally demanding and resulted in dual-task interference. This finding may have clinical implications as it suggests a relationship between gait synchronization and attentional resources. For example, walking while performing verbal tasks has been shown to be related to motor abilities in older adults, and physical rehabilitation targeting balance and gait deficits may lead to improvements in this population (Hall et al., 2011). The addition of haptic communication may lead to accelerated improvements in this population due to an increase in available sensory information, but this will need to be addressed in future research.

**FIGURE 2:**
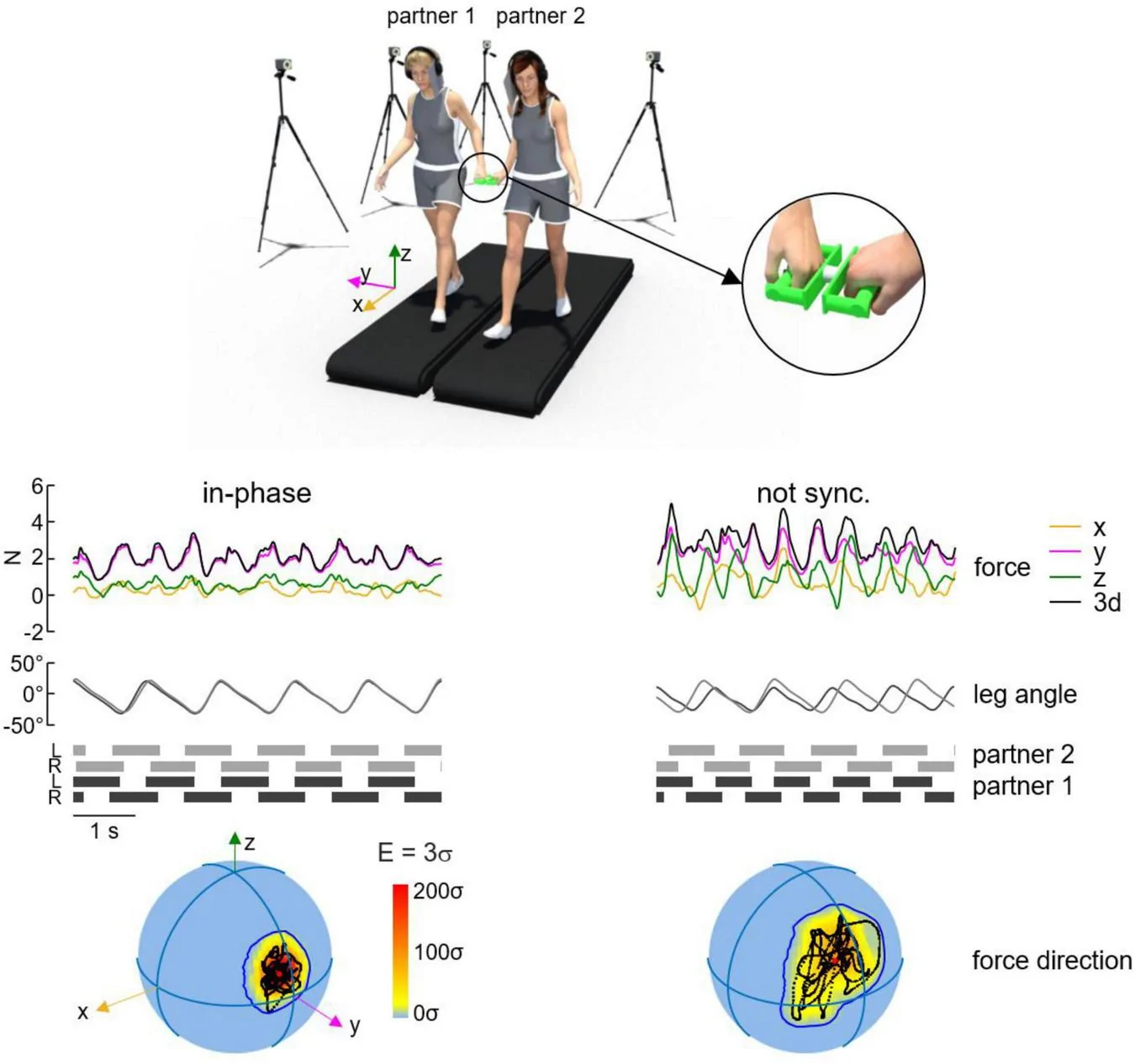
Variability of spatial orientation of interaction forces during walking hand in hand. Representative interaction forces and arm/leg kinematics during non-synchronized and in-phase walking over five consecutive strides. From top to bottom: interaction forces (x, y, z, and 3d), contact side leg angle and lower limbs’ stance durations for each partner. At the bottom: spherical spatial density of the force vector directions during the corresponding five consecutive strides. The color scale indicates density diagrams calculated using the Kamb method for directional data. Note the increased variability of the force direction sphere during non-synchronized walking when compared to in-phase. Figure adapted from Sylos-Labini et al. (2018).

The magnitudes and directions of the interaction forces between hands are likely important for the haptic communication between paired individuals. These forces are generally less than 5 N (Sawers et al., 2017; Sylos-Labini et al., 2018; Wu et al., 2021, 2024), and are too small to generate significant mechanical changes to one’s walking pattern. Sylos-Labini et al. (2018) used an instrumented handle to quantify the interaction forces between pairs of participants (Figure 2). They were able to determine that the lateral component of the interaction force (related to arm abduction or the participants) was ∼2–3 N larger than the other components, while the oscillations of forces along all three axes were relatively similar in magnitude during walking and were less variable during synchronized stepping between pairs. Moreover, shoulder muscle activity did not differ during hand holding when compared to walking alone (Sylos-Labini et al., 2018), consistent with the speculative suggestion that the neural coupling between cervical and lumbosacral pattern generation circuitries governing quadrupedal coordination is conserved (Zehr et al., 2016). Thus, step synchronization appeared to emerge from reduced variability in the force vectors. Indeed, unintentional frequency locking of steps was frequently observed in pairs walking with hand contact when only haptic information was available, while visual and auditory cues were obstructed. Taken together, this evidence highlights that these haptic forces provide cues to paired participants who are unconsciously used to coordinate step patterns during walking, as opposed to mechanical forces dictating movement changes between pairs. These results (Figure 2) also raise an intriguing question about the involvement of sophisticated neural mechanisms capable of detecting and adapting to subtle variations in force vector patterns to support interactive locomotor coordination.

The haptic communication occurring between pairs of individuals holding hands can also be used to provide non-verbal assistance for locomotor tasks. For instance, research has shown that haptic interaction forces of slightly larger magnitude than hand holding (10–30 N) are sufficient to provide guidance to coordinate locomotor patterns between leaders and followers (Sawers et al., 2017), and between an expert dancer and an untrained individual during a paired stepping task (Wu et al., 2024). When walking on a beam, participants were able to walk further without falling, reduced lateral sway, and decreased angular momentum in the frontal plane when low levels of haptic assistance (< 5 N) were provided (Wu et al., 2021). These studies lead to the conclusion that the direction of the haptic interaction forces may encode gait changes between pairs (Wu et al., 2024) and provide temporal cues (Sawers et al., 2017) to facilitate locomotor changes.

There is currently a gap in the literature regarding the advantages and disadvantages of differing hand holding configurations, such as palm-to-palm or interlacing finger haptic interactions. Hand positioning has been suggested to be of importance during guidance of people with visual impairments. For instance, hand-over-hand manipulation tends to be more passive for the individual, while hand-under-hand is less controlling and facilitates increased haptic exploration during hand guidance tasks (Miles, 1997). Investigations into the performance of haptic tasks have also indicated increased performance when multiple fingers are used (Morash et al., 2013, 2014). Current experimental paradigms that examine force transmission during human-human haptic communication involve participants holding handles that are more similar to palm-to-palm interactions as opposed to interlacing fingers. It would be interesting to examine different hand-holding configurations to explore if one is beneficial over the other and enhances the quality of the haptic information transferred between partners, and whether there are clinical implications for these findings.

## Developmental aspects

Haptic interaction with objects and people has a critical role also in the development of locomotion at an early age. Strikingly, toddlers who just started walking independently prefer to do so while carrying objects than with hands free (Karasik et al., 2011). This behavior also has a social significance, as shown by the fact that when toddlers walk toward their caregivers, they often bring objects to share. Even crawlers, who did not start walking independently, often carry objects (Karasik et al., 2011). The added task of object transport increases the complexity and cost of motor coordination, but infants selectively choose lighter objects to carry and explore (Heiman et al., 2019).

Haptic exploration of the environment by toddlers during locomotion does not only involve the hands but can even be performed by the foot. Thus, it has been shown that, in contrast with adults, toddlers often place their feet onto an obstacle or across the edges of the stairs when stepping with the clear intent of obtaining knowledge of the object properties (Dominici et al., 2010).

With regards to hand-to-hand interactions during walking, it has recently been shown that children (6–8 years old) exchange haptic communicative forces with the partner (whether an adult or another child) significantly different from those produced by dyads of adults only: children tend to be more compliant in the interaction than adults (Avaltroni et al., 2025). Spontaneous synchronization of locomotion in child-child and adult-child dyads as received less attention in the current literature, but this may be difficult to quantify given the variable spatiotemporal walking patterns observed in children (Rygelová et al., 2023). Since haptic interactions are integral for movement and social development, it would be interesting to explore longitudinal responses of child-child and adult-child walking and their influence on child development.

## Technological advances: human-robot haptic interactions

**FIGURE 3:**
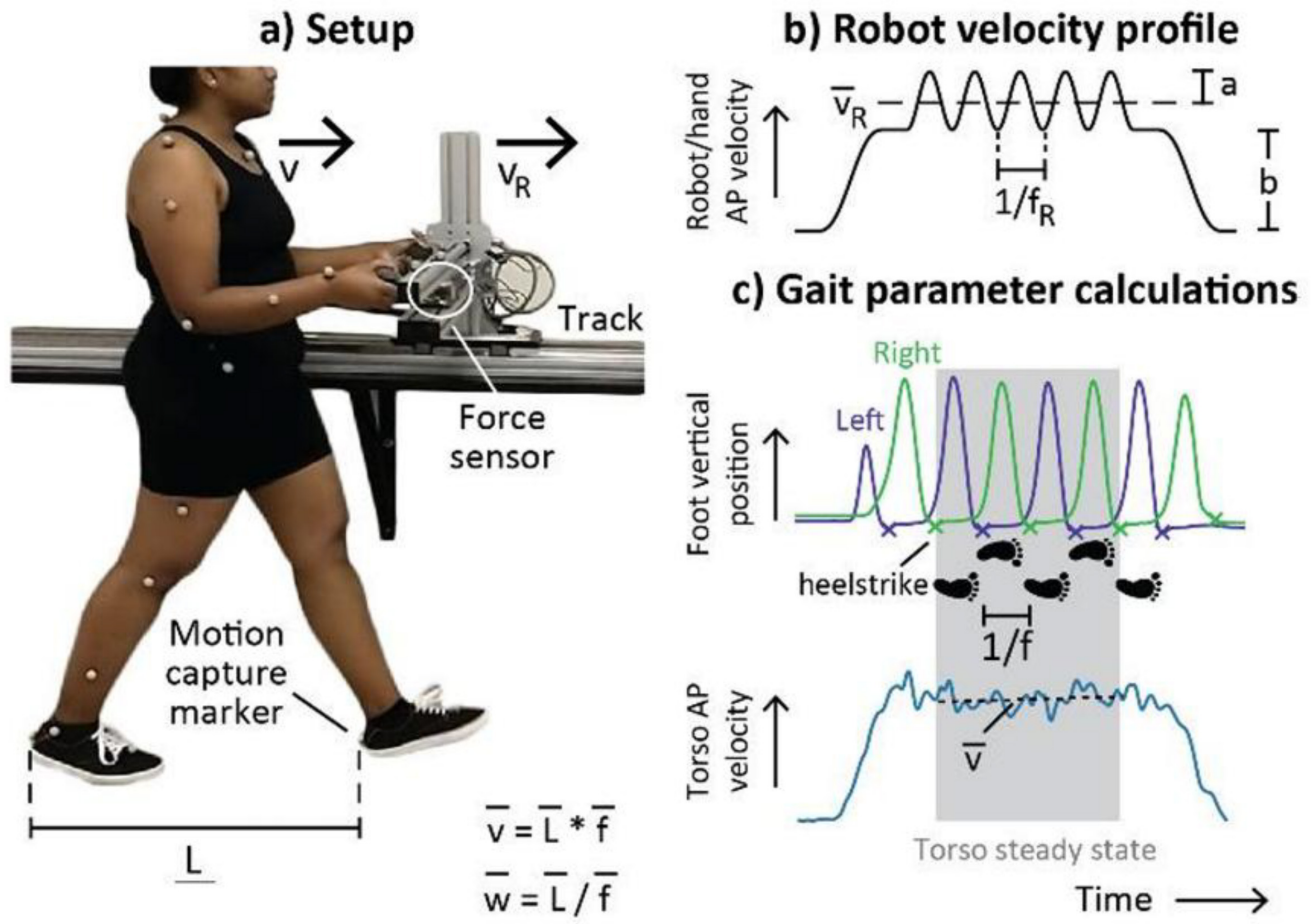
Locomotor adaptations during human-robot haptic interactions. **(a)** Participants walked forwards while holding the robot (“Slidey”) handles. A sensor embedded in the robot measured human-robot interaction forces. Motion capture data were used to calculate mean human velocity (v), step length (L), and step frequency (f) during steady-state walking. **(b)** The human-robot interaction controller consisted of transient velocity pulses at frequency f_R_ and amplitude a superimposed on a trapezoidal velocity profile with bias b. We predicted that the robot’s mean velocity v_R_ would affect human velocity while robot pulse frequency f_R_ would affect human step frequency. **(c)** Mean gait parameters were calculated during four steps when mean human torso velocity *v* was constant. Note the synchronization between the robot oscillations and vertical foot position. Figure from Wu and Ting (2025) (reproduced with permission).

Recent technological advances have resulted in the development of robots that provide a haptic link to participants through their hands, leading to adaptive locomotor responses. For example, robots such as Mako-no-te (Hasegawa and Okada, 2019), Ophrie (Regmi et al., 2022a,b), and Slidey (Wu and Ting, 2025) guide human overground locomotion through a haptic interaction at the participant’s hands. Evidence has shown that the interaction forces between humans and these robots is ∼5 N (Regmi et al., 2022a,b), similar to the forces observed during human-human hand holding haptic interactions. Ophrie is a wheeled robot designed to lead participants side-by-side and provides walking assistance through a mechanical arm that participants hold (Regmi et al., 2022a,b). Studies conducted with Orphie indicated that participant arm stiffness was important during human-robot interaction, whereby arm stiffness was ∼22% lower when participants were not aware of direction changes in the robot, and these arm stiffness changes likely facilitate better haptic communication during the walking task (Regmi et al., 2022a,b). Wu and Ting (2025) designed a robot “Slidey” with two handles that provide velocity pulses that influence participant gait velocity and step frequency changes through haptic interaction (Figure 3). However, the influence on participant gait patterns only occurred when participants were instructed to match their step timing to the pulses provided by the robot. The haptic interaction forces between the participant and robot were close to zero, providing evidence that the gait characteristic changes were due to haptic sensorimotor engagement as opposed to propulsion by the robot. These robots provide evidence of gait changes in health adults, highlight the importance of arm compliance to optimize haptic interactions, and provide support for clinical use for locomotor rehabilitation.

## Clinical implications for haptic interactions

The use of upper limb haptic interactions has the potential to reveal impairments and provide additional information about corrupted sensorimotor neural processes, as well as complement the rehabilitation of locomotion and adaptive locomotion. For instance, upper limb compliance related to dynamic muscle tone is functionally significant in locomotor control (Bernstein, 1940), and is of utmost importance during the performance of interactive locomotion (Dolinskaya et al., 2023). Altered upper limb muscle tone may affect the quadrupedal coordination of locomotor tasks, and haptic interactions may be useful in the clinical assessment of the extent upper limb muscle tone impairments on locomotor control (Cacciatore et al., 2024).

Pathologies affecting the sensorimotor control of the upper limbs such as diabetic neuropathy, stroke, cerebellar ataxia, and cerebral palsy could potentially benefit from haptic training during rehabilitation. For example, past work has shown that an anchored railing placed next to a treadmill providing an opportunity for the hand to provide small levels of force can enhance stability in healthy (Dickstein and Laufer, 2004) and pathological (Oates et al., 2017) groups. Similarly, collective transport of a light object, patient-clinician hand holding, and patient-robot interactions likely provide similar support to a fixed support structure but facilitate overground locomotion in the patient as opposed to treadmill walking. This haptic information could improve safety during rehabilitation and ultimately increase the distance traveled by the patient, therefore aiding in locomotor endurance, as well as maximize outcome potentials during rehabilitation.

It is worth stressing that there is a growing interest in investigating the rehabilitation of adaptive locomotion, since walking impairments may only present themselves during the performance of locomotor tasks and be absent from basic walking (Cappellini et al., 2018). Haptic communication and interactive locomotion are examples of adaptive locomotion with physiological relevance. The addition of object transport to rehabilitation programming may provide opportunities to challenge the management of attentional resources devoted to locomotion, arm-leg coordination during adaptive locomotion, as well as the control of arm compliance to enhance the perception of haptic information. Recent work has illustrated the benefits of anticipatory control training in older adults. For instance, research has examined training older adults and people with stroke using tasks that involve necessary anticipatory postural adjustments, such as pushing a medicine ball hanging from a ceiling (Curuk et al., 2020) or catching a medicine ball (Jagdhane et al., 2016). These programs resulted in earlier generation of anticipatory postural adjustments in the training groups, potentially leading to enhanced balance control in the training groups (Aruin, 2016). These clinical studies indicate that training anticipatory control in older adults and in pathological populations can be beneficial to motor control. Taking this into consideration, similar haptic training using object transport may help improve the coordination of grip control and absorption of impact forces during this task. A training protocol that facilitates real-time feedback of a carried object along with a target range to maintain object movement (for instance, having a laser pointer attached to the object and projected at a wall in front of the participant, with a target box on the wall in which the participant must maintain the laser pointer) could lead to enhanced coordination of this task. Training while transporting objects of different sizes and weights may also help rehabilitate the adaptation of one’s locomotor body scheme used in the anticipatory control of upper limb mechanics in preparation for heel contact.

Given human-human interactive locomotion is related to the appearance of spontaneous synchronization of step patterns, patient-clinician handholding may provide an opportunity to unconsciously train targeted spatiotemporal gait characteristics. Also, since forced gait synchronization has been shown to be less beneficial to adapting asymmetric gait patterns between participants (Nessler et al., 2015), these non-verbal mechanisms may be more beneficial for rehabilitation. However, the clinical benefits of human-human haptic communication remain to be explored. Recent research has documented the benefits of rhythmic auditory stimulation on the reacquisition of asymmetric temporal patterns in people with stroke (Lee et al., 2018) and Parkinsons disease (Ghai et al., 2018). Moreover, vibratory rhythmic haptic cueing of the upper arms has been shown to improve walking speed and arm-leg coordination in older adults (Khiyara et al., 2025). Handholding between a clinician and patient could provide similar rhythmic information to patients or could even be used to augment rhythmic auditory stimulation therapy to enhance improvements in gait symmetric in patients, however, future research will need to address these possibilities. Finally, Wu and Ting (2025) suggested patient-robot haptic interactions may be useful in training human-robot collaborative tasks, collective object transportation, and ultimately influence training of step length and timing in pathological populations. While there appears to be promising research that suggests haptic communication is beneficial for clinical integration, some limitations should be addressed. First and foremost, it should be noted that the research supporting links between haptic communication and locomotor rehabilitation is relatively new, and further work is needed to understand the potential benefits its use, as well as to understand the mechanisms relating to locomotor function. Current research has focused primarily on human-human and human-robot haptic interactions in healthy younger adults, and future work on the clinical translation of these activities in a variety of pathological populations must be examined. Moreover, it is likely that a minimal level of locomotor function is required to exploit these benefits in a clinical setting. For example, a patient with a very low and irregular gait speed may not benefit as much from additional haptic information. However, current research supports further exploration of this area to understand the full potential of haptic communication for clinical use.
