# Correction: Sophisticated use of upper limb haptic interactions during adaptive locomotion

**DOI:** 10.3389/fpsyg.2026.2031409

**Published:** 2026-10-27

**Authors:** Michael J. MacLellan, Yury Ivanenko, Priscilla Avaltroni, Francesco Lacquaniti, Francesca Sylos-Labini

**Affiliations:** 1 Department of Applied Human Sciences, University of Prince Edward Island, Charlottetown, PE, Canada; 2 Laboratory of Neuromotor Physiology, Istituto di Ricovero e Cura a Carattere Scientifico Fondazione Santa Lucia, Rome, Italy; 3 Department of Systems Medicine and Center of Space BioMedicine, University of Rome Tor Vergata, Rome, Italy

**Keywords:** sensorimotor control, haptic communication, interaction forces, quadrupedal coordination, interactive locomotion

In the published article, the grant code CUP J83C22002060007 was omitted from the **Funding** statement. The correct **Funding** statement reads:

“The author(s) declared that financial support was received for this work and/or its publication. This work was supported by the Italian Ministry of Health and funded by the European Union - Next Generation EU - NRRPM6C2 - Investment 2.1 Enhancement and strengthening of biomedical research in the NHS, PNRR-POC-2022-12375705, CUP J83C22002060007.”

The original version of this article has been updated.

